# Roles of Small Noncoding Vault RNAs in Intestinal Epithelium Homeostasis and Diseases

**DOI:** 10.3390/ijms262311565

**Published:** 2025-11-28

**Authors:** Amy VanderStoep, Lan Xiao, Jian-Ying Wang

**Affiliations:** 1 Cell Biology Group, Department of Surgery, University of Maryland School of Medicine, Room S243, HSF-II, 20 Penn St., Baltimore, MD 21201, USA; avanderstoep@som.umaryland.edu (A.V.); lxiao@som.umaryland.edu (L.X.); 2 Department of Pathology, University of Maryland School of Medicine, Room S243, HSF-II, 20 Penn St., Baltimore, MD 21201, USA; 3 Baltimore Veterans Affairs Medical Center, Baltimore, MD 21201, USA

**Keywords:** noncoding RNAs, vault RNAs, RNA-binding proteins, posttranscriptional regulation, intestinal mucosal growth, gut permeability, inflammatory bowel disease (IBD), critical surgical disorders

## Abstract

The mammalian intestinal epithelium is a rapid self-renewing tissue that functions as a physical barrier against a wide array of noxious substances and the gut microbiome that inhabit the intestinal lumen. Homeostasis of the intestinal epithelium is tightly regulated via well-controlled mechanisms and depends on rapid gene expression pattern alterations to effectively regulate cell survival, adapt to stress, and keep epithelial integrity in stressful environments. Vault RNAs (vtRNAs) are small noncoding RNAs that are highly expressed in the intestinal epithelium and involved in many cellular processes essential for healthy and pathological states. In this review, we provide a broad discussion of vtRNA biogenesis, the roles of vtRNAs in intestinal mucosal renewal and gut barrier function regulation, and the interactions of vtRNAs with RNA-binding proteins in modulating mRNA stability and translation. We also highlight the implications of vtRNAs in human gut mucosal disorders and point out vtRNAs as potential biomarkers and/or novel targets for developing new diagnostic and/or therapeutic modalities for identifying and preserving the integrity of the intestinal epithelial lining in patients with critical disorders.

## 1. Introduction

The gastrointestinal tract is a complex series of organs extending from the mouth to the anus. A large portion of that is the intestine, which is primarily responsible for food digestion and absorption of water and nutrients. The epithelium of the mammalian intestinal mucosa is lined by a single layer of columnar epithelial cells that serve as a dynamic barrier, allowing necessary substances to be absorbed, while simultaneously serving as a barrier to the harmful substances, such as luminal toxins or bacteria [1]. The intestinal epithelium is a tissue that is constantly self-renewing in the body. Its homeostasis is preserved via tightly controlled processes that depend on intestinal epithelial cells (IECs) to regulate proliferation, apoptosis, migration, differentiation, and cell-to-cell interactions [2,3,4]. The crypt base is the site of continuous replication of intestinal stem cells (ISCs) and amplified progenitor cells. This process drives epithelial renewal. Meanwhile, the newly divided cells migrate toward the gut lumen along the crypt-villus axis, differentiating into various mature cell types. Apoptosis occurs in the crypt regions of the intestine to counterbalance cell proliferation and at the luminal surface of the mucosa, where mature cells are lost. Nonetheless, dysregulated homeostasis of the intestinal mucosa occurs commonly in many pathologies and human diseases, including inflammatory bowel disease (IBD) and various critical surgical disorders such as sepsis, shock, severe trauma, massive thermal injury, and emergent surgical operations [5]. Disruption of the intestinal epithelial integrity leads to the translocation of luminal substances and bacteria into the bloodstream and, in some instances, can cause multiple organ system failure and death.

Studies using high-throughput transcriptomics reveal that the majority (>96%) of the mammalian genome is transcribed into a huge number of noncoding RNAs (ncRNAs). Many studies have identified these ncRNAs as crucial participants and regulators in diverse cellular pathways and human pathologies [6,7,8]. Conversely, protein-coding sequences only account for a minority (<4%) of the cellular transcriptional output. Generally categorized as either small (<200 nucleotides) or long (>200 nucleotides), these ncRNA molecules can serve both housekeeping and regulatory functions. The roles and mechanisms of ncRNAs in the regulation of intestinal epithelial homeostasis and human diseases are particularly evident for long ncRNAs (lncRNAs) and microRNAs (miRNAs) [6,9]. However, recent studies show that small noncoding vault RNAs (vtRNAs) are a novel class of biological regulators of the intestinal epithelial function and participate in many aspects of gut mucosal pathophysiology [10,11,12,13]. In this review, we highlight the importance of vtRNAs in the control of intestinal epithelial renewal and barrier function and discuss the molecular mechanisms by which vtRNAs regulate gene expression at the posttranscriptional level via interactions with RNA-binding proteins (RBPs). We also point out the implications of altered vtRNAs in the pathogenesis of intestinal mucosal inflammatory/injury, gut barrier dysfunction, and other diseases.

## 2. vtRNAs Biogenesis and Function

vtRNAs are small (~100 nucleotide) ncRNAs that are transcribed by RNA polymerase III and highly conserved across mammalian genomes. vtRNAs were initially isolated in the 1980s from rat liver tissue. Small ovoid bodies found in the samples were purified and found to be distinctly structured proteins, referred to as vaults due to their “cathedral vault”-like structures seen under electron microscopy. Simultaneously, a species of small RNAs with unique composition were also discovered. The base composition of these RNAs was one component that made them unique, with researchers noting 12% adenosine, 29.7% guanine, 30.9% uridine, and 27.4% cytidine. It is the relatively low proportion of adenosine that separated these RNAs from similarly sized and structured RNAs such as U8, 5S, or 5.8S RNA in rats [14]. Further analysis and characterization reveal that both the major vault proteins (MVPs) and the loci of these vault-associated RNAs are highly conserved among eukaryotes, although many mammals have fewer paralogs of vtRNAs [15,16]. Vaults are the giant cytoplasmic ribonucleoprotein (RNP) particles made up of a MVP and two minor vault proteins, vPARP and TEP1 [11,17]. Multiple vtRNAs can be associated with each vault, with early rat studies showing ~9 RNA molecules associating with each vault [14]. While originally thought to be always incorporated with vaults and other protein molecules, it is now widely accepted that these vtRNAs hold other important functions as independent molecules, free from the vault complexes. In fact, only about 5% of vtRNAs are associated with vault proteins in normal biological conditions, whereas the remaining 95% of vtRNAs are free molecules distributed throughout the cytoplasm [18,19,20,21]. Extensive work has been done to elucidate the structure and function of both these vault proteins and their associated vtRNAs. Utilizing microscopy and reconstruction techniques, it was hypothesized that the vtRNAs are likely associated at the ends of the vault caps, not the central barrel component [20]. Additionally, the TEP1 subunit has been shown to be required for the stable association of the vtRNA to the vault particle, specifically the p80 homology region [22,23].

**Figure 1:**
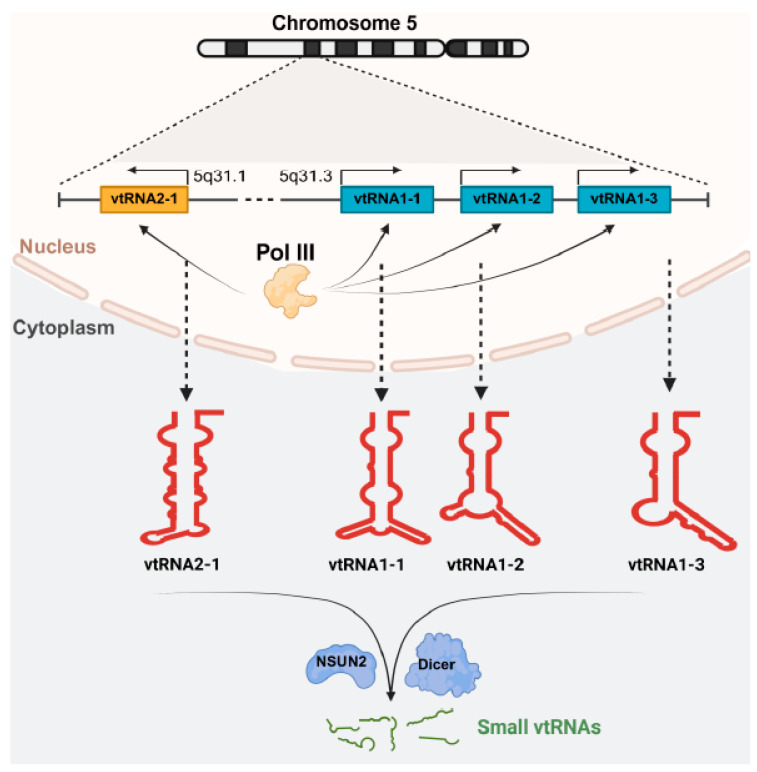
vtRNA biogenesis in human tissues. Four vtRNAs, including *vtRNA1-1*, *vtRNA1-2*, *vtRNA1-3*, and *vtRNA2-1*, are transcribed from two loci on chromosome 5 by RNA polymerase III (Pol III). Locus 5q31.3 encodes *vtRNA1-1*, *vtRNA1-2*, and *vtRNA1-3*, while locus 5q31 encodes *vtRNA2-1* only. After transcription, vtRNAs are associated with vault particles or free molecules in the cytoplasm. vtRNAs can also undergo fragmentation into small vtRNAs via NSUN2 (NOP2/Sun RNA Methyltransferase 2) and/or Dicer-dependent pathways.

Humans produce 4 vtRNA paralogues, *vtRNA1-1*, *vtRNA1-2*, *vtRNA1-3*, and *vtRNA2-1*, which are located on chromosome 5q31 in two loci and are all transcribed by RNA Polymerase III (Figure 1). Humans also have two vtRNA pseudogenes, vtRNA2-2p and vtRNA3-1p, that are located on chromosomes 2p14 and Xp11.2 [16,24]. *vtRNA1-1*, *vtRNA1-2*, *vtRNA1-3*, and *vtRNA2-1* exhibit distinct secondary structures with A-box and B-box internal promoters [21,25,26]. There are conserved ‘panhandle’ structures with a 25–30 nucleotide-long stem and central domain of variable size and sequence in all four human vtRNAs [11]. On the other hand, rats and mice only express a single vtRNA that is ~144 nucleotides long and exhibits a similar structure to human *vtRNA1-1* [25,26]. In addition, vtRNA and its association with the TEP1 vault component are also identified in *Trypanosome brucei*, a parasite that causes African sleeping sickness [27,28].

Moreover, vtRNAs can be further processed into various small fragments, named as small vault RNAs (~22–24 nucleotide), via NSun2-mediated methylation and Dicer protein [11,29,30,31]. The biogenesis of small vtRNA fragments can be a Dicer-dependent manner, but it is independent of Drosha complexes [11,32]. Although overall function of these small fragments of vtRNAs remains largely unknown, some small vtRNAs act as miRNAs by binding to argonaute proteins and assisting in the processes of keratinocyte progenitor cell transcription [11,33,34].

vtRNAs are involved in many aspects of cellular processes such as mRNA splicing, nuclear transport, drug resistance, synaptogenesis, lysosome function, apoptosis, viral replication, and tumorigenesis [11,17,35,36]. The four human vtRNAs have distinct biological functions via different targets and mechanisms, though they differ only slightly in primary and second structures. For examples, knockdown of *vtRNA1-1* elevates levels of apoptotic cell death during prolonged starvation in HeLa cells, but *vtRNA1-3* silencing does not affect rate of the apoptosis. Further study shows that *vtRNA1-1* knockout disrupts the Pi3K/Akt pathway as well as the ERK1/2 cascade, and these changes are reliant on a short stretch of 24 nucleotides in the *vtRNA1-1* central domain [37]. *vtRNA1-1* also regulates autophagy by associating with RBP p62 [38], whereas *vtRNA2-1* functions as a tumor suppressor by interacting with protein kinase R in a wide range of cancer cells [39,40]. A recent study using a mouse genetic model shows that loss-of-function of vtRNA is compatible with relatively normal development and survival, but several connections to physiology and pathophysiology have been uncovered [41]. Emerging evidence also indicates that human vtRNAs play crucial roles in drug resistance, apoptosis, cancers, and neurologic disorders such as Parkinson’s disease and epilepsy [11,17]. As more functions of these vtRNAs are exposed, it begs the question of how many other pathways these unique molecules influence the physiological tasks of human cells. Additionally, there is a question of how many pathological and disease processes are influenced by both the function and dysfunction of vtRNAs.

## 3. vtRNAs in Intestinal Epithelium Homeostasis

The intestinal space is directly exposed to a wide array of luminal noxious substances and colonized by trillions of commensal bacteria. The intestinal epithelium separates these luminal substances and microorganisms from sterile tissue. In response to stressful environments, maintenance of the intestinal epithelial integrity requires epithelial cells to rapidly alter gene expression patterns that lead to adjustments in cell proliferation, migration, apoptosis, differentiation, and cell-to-cell interactions [3,42,43]. Although gene expression programs that control the intestinal epithelial integrity are strongly regulated at the transcriptional level, an increasing body of evidence indicates that posttranscriptional events, particularly altered mRNA stability and translation by ncRNAs and RBPs, play an essential role in regulating the intestinal mucosal homeostasis [44,45,46]. The roles and mechanisms of miRNAs, lncRNAs, and RBPs have been extensively investigated and comprehensively summarized in several review articles [2,7,44,47]. However, vtRNAs have recently joined the list of molecules that have an impact on intestinal epithelium homeostasis [10,47], although the exact mechanisms by which vtRNAs regulate biological function of epithelial cells remain to be fully elucidated.

### 3.1. vtRNAs in Intestinal Mucosal Renewal

The human intestinal epithelium goes through ~10^11^ mitoses daily, and the epithelium constantly renews throughout a human’s lifetime. The rapid and dynamic turnover rate of the intestinal epithelium is driven by ISCs and tightly regulated by numerous intracellular and extracellular factors at multiple levels [48,49]. To examine the function of *vtRNA1-1* in the intestinal epithelium, our group utilized a transgenic gain-of-function approach to generate a mouse model that specifically expressed human *vtRNA1-1* (vtRNA1-1Tg) in the intestinal epithelium under control of the A33 promoter [12]. As expected, vtRNA1-1Tg mice exhibit specific *vtRNA1-1* overexpression in the intestinal mucosal tissues, but this same overexpression is not seen in heart, lung, liver, kidney, or spleen tissue. Transgenic expression of *vtRNA1-1* in mice does not affect the levels of endogenous *vtRNA* in the intestinal mucosa. Importantly, vtRNA1-1Tg mice display inhibition of growth within the small intestinal mucosa. This is evident in the decreased cell proliferation within the crypts and reduction in crypt and villus size. Further evaluation of these reductions shows that the lengths of crypts and villi in the small intestinal mucosa of vtRNA1-1Tg mice decrease by ~25% and ~35%, respectively. Increasing the levels of cellular *vtRNA1-1* in the intestinal epithelium also causes defects in Paneth cells that provide multiple secreted and surface-bound niche signals and are essential for ISC proliferation [46,48,49]. Lysozyme-positive Paneth cells are located at crypt bases in control littermate mice, but Paneth cell numbers are observed to be significantly reduced (~36%) in the small intestinal mucosa of these vtRNA1-1Tg mice. On the other hand, increasing the levels of *vtRNA1-1* levels in the intestinal mucosa fails to alter enterocyte differentiation and does not inhibit growth of colonic mucosa.

We also examined the effect of *vtRNA1-1* on renewal of the intestinal epithelium in an ex vivo model using the primary culture of intestinal organoids generated from vtRNA1-1Tg and littermate control mice [12]. Although intestinal organoids derived from both littermate and vtRNA1-1Tg mice consist of multiple buds and cells on day 4 after initial culture, the organoids from vtRNA1-1Tg mice display much slower growth compared to those from littermate mice, since there is a significant decrease in BrdU incorporation and surface area of organoids generated from vtRNA1-1Tg mice compared to those derived from control littermates. Consistent with the observation in vivo, intestinal organoids derived from vtRNA1-1Tg mice also exhibit decreased numbers of Paneth cells. Since these primarily cultured intestinal organoids lack stromal, immune, and neural cells [50,51], these results obtained from intestinal organoids ex vivo indicate that the inhibited renewal of intestinal epithelium seen in vtRNA1-1Tg mice is more likely resulting from an autonomous regulatory effect of this vtRNA as the primary mechanism, and not from the effects of externally secreted factors.

Like *vtRNA1-1*, elevation of the *vtRNA2-1* levels also inhibits the growth of intestinal organoids [10]. Ectopically expressing *vtRNA2-1* inhibits DNA synthesis in multiple cells and reduces the sizes of organoids ex vivo. Moreover, *vtRNA2-1* overexpression leads to Paneth cell defects in intestinal organoids, since the number of lysozyme-positive cells decreases markedly in the organoids transfected with *vtRNA2-1* expression vector relative to those transfected with control vector. In agreement with the results from an ex vivo system, *vtRNA2-1* overexpression by infecting mice with a lentiviral vector expressing *vtRNA2-1* (lenti-vtRNA2-1) also inhibits growth of the small intestinal mucosa, as demonstrated by a significant decrease in the proliferating crypt cell population in lentiviral-vtRNA2-1-infected mice compared with animals infected with control vectors. The number of BrdU-positive cells in crypts of the intestinal mucosa decreases by ~85% in lenti-vtRNA2-1-infected mice compared with controls.

Unexpectedly, increasing the levels of *vtRNA2-1* does not affect Paneth cell function in the small intestine of mice, since there are no detectable changes in the number of lysozyme-positive cells in the mucosa between lenti-vtRNA2-1-infected mice and mice infected with control vector. Unlike most IECs that are rapidly turned over within a few days, Paneth cells can persist for months in healthy individuals after maturation [48,52]. In primarily cultured intestinal organoids ex vivo, however, Paneth cells develop quickly with organoid growth within days after primary culture [46,53]. The differences in the response of Paneth cells to *vtRNA2-1* overexpression between ex vivo and in vivo model systems suggest that *vtRNA2-1′s* role is more in the inhibition of Paneth cell development in the small intestinal mucosa but perhaps only has a more minor role in regulating the activity of already differentiated Paneth cells. Taken together, these findings obtained from transgenic mice and primarily cultured intestinal organoids strongly support the notion that vtRNAs, specifically *vtRNA1-1* and *vtRNA2-1*, are potent inhibitors of intestinal epithelial renewal and Paneth cell function.

In support of the role of vtRNAs in intestinal mucosal growth, *vtRNA1-1* is also involved in the regulation of epidermal renewal in human skin and other tissues [54,55]. An RNA methyltransferase (Misu/Nsun2) is identified as a requirement for balancing stem cell renewal and differentiation in skin cells. It is well understood that RNA modifications influence their metabolism and function. NSUN-mediated methylation of cytosine in *vtRNA1-1* occurs with frequency in human cells. This study also discovered that *vtRNA1-1* interacts with the serine/arginine-rich splicing factor 2 (SRSF2), a novel *vtRNA1-1* binding protein. SRSF2 directly binds to the non-methylated *vtRNA1-1* with much higher affinity, affecting the production of distinct small vtRNAs (svtRNAs). It is these svtRNAs that regulate epidermal differentiation [55]. *vtRNA1-2* and its subsequent svtRNA product are also implicated in cellular proliferation. Dicer-dependent svtRNA1-2 is associated with Argonaute 2 protein; unlike its precursor *vtRNA1-2* that predominates in the cytoplasm, svtRNA1-2 localizes in the nucleus. The expression levels of several genes necessary for cell migration, motility, proliferation, and growth in skin and other tissues are also altered by changes in cellular *vtRNA1-2* levels, while deletion of *vtRNA1-2* and subsequent reduction in svtRNA1-2 lead to impaired cell proliferation [56].

### 3.2. vtRNAs in Gut Barrier Function

**Figure 2:**
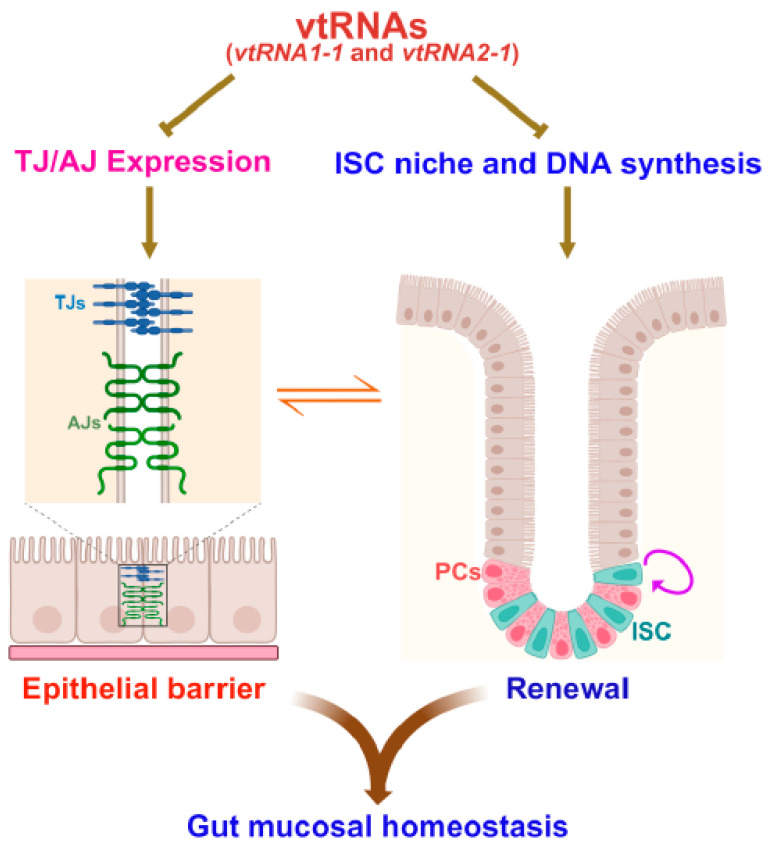
vtRNAs regulate homeostasis of the intestinal epithelium by altering constant mucosal renewal and gut barrier function. Increased vtRNAs inhibit growth of the intestinal mucosa via a decrease in Paneth cell/ISC niche activity and disrupt the epithelial barrier function by repressing the expression of TJ/AJ proteins.

The intestinal epithelial barrier comprises specialized structures consisting of various intercellular junction proteins, including TJs and adherens junctions (AJs), that surround the subapical region of epithelial cells (Figure 2). The TJ is the most apical element of the junctional complex that establishes a selectively permeable barrier and prevents most molecules, even small ones, from leaking between cells [1,57,58,59]. TJ complexes are primarily composed of transmembrane TJ proteins. These include occludin and members of the claudin family. These proteins also interact with a cytosolic plaque of other TJ proteins like ZO-1 that are associated with the cytoskeleton [1,57]. Below the TJs are the cadherin-rich AJs. These mediate intercellular adhesion and play important roles in assembling and modulating the epithelial barrier. The formation of TJ and AJ complexes is a very dynamic process. Involved cellular proteins undergo continuous turnover and remodeling at a rapid pace, which is tightly controlled by various factors and signals at multiple levels [1,57,58]. Several intestinal epithelium-enriched ncRNAs, including miRNAs and lncRNAs, are involved in regulating gut barrier function and have been nicely summarized [2,57,58].

Recently, emerging evidence indicates that vtRNAs are biological regulators of gut barrier function primarily by altering the levels of TJ and AJ proteins [10,12,60]. In studies conducted in an in vitro model for intestinal epithelial barrier using cultured human IECs, ectopically expressed *vtRNA2-1* specifically inhibits expression of TJs claudin-1 and occludin and AJ E-cadherin without effect on the expression levels of TJs ZO-1 or JAM-A, while increasing the levels of *vtRNA1-1* decreases the expression levels of TJs, including claudin-1, claudin-2, claudin-3, claudin-7, occludin, JAM-A, and ZO-2. It does not appear to alter E-cadherin expression levels. Importantly, elevation of either *vtRNA2-1* or *vtRNA1-1* level disrupts the epithelial barrier function. This is shown by a decrease in transepithelial electrical resistance (TEER) values alongside an increase in the paracellular flux of FITC-dextran. In contrast, silencing *vtRNA2-1* or vtRNA1-1 increases the expression of TJ proteins and enhances epithelial barrier function, since vtRNA silencing increases TEER and decreases paracellular flux of FITC-dextran.

In an ex vivo model consisting of intestinal organoids, elevation of the levels of *vtRNA2-1* ortholog also reduces the levels of claudin-1, occludin, and E-cadherin without the effect on ZO-1 content, as measured by immunostaining assays [10]. In the control group, claudin-1 and occludin clearly line the basolateral and apical membrane regions, with slight cytoplasmic staining present in some organoids. E-cadherin predominantly lines the basolateral membrane areas and is noticeably absent from the cytoplasm and apical region of cells, while ZO-1 is limited primarily at or near the apical membrane. Transfection of intestinal organoids with the *vtRNA2-1* expression vector increases the levels of *vtRNA2-1*, although it does not alter the levels of endogenous mouse *vtRNA* or *U6 RNA*. Consistent with the observations in cultured IECs in vitro, ectopically expressed *vtRNA2-1* also inhibits expression of claudin-1, occludin, and E-cadherin, as shown by a significant decrease in the levels of immunostaining intensity of claudin-1, occludin, and E-cadherin in organoids transfected with a *vtRNA2-1* expression vector relative to organoids transfected with control vector. Although *vtRNA2-1* overexpression does not alter ZO-1 levels or its subcellular distribution in intestinal organoids.

Furthermore, exposure of intestinal organoids to vtRNA-rich extracellular vesicles (EVs) increases organoid paracellular permeability [12]. In this study, intestinal organoids were seeded in a basement matrix, embedded in standard growth medium for 2 days, and then cultured in medium containing EVs. FITC-dextran was microinjected into the organoid lumen 24 h after administering EVs, and paracellular permeability was examined at different times after microinjection. Treatment of intestinal organoids with vtRNA-enriched EVs led to increases in paracellular permeability, demonstrated by rapidly decreasing luminal FITC intensity in organoids exposed to *vtRNA1-1*-rich EVs, compared to controls.

In experiments conducted in vtRNA1-1Tg mice, intestinal epithelium-specific transgenic expression of *vtRNA1-1* inhibits the expression of small intestinal mucosal TJ proteins, including claudin-1-3, claudin-7, occludin, JAM-A, and ZO-2, and AJ E-cadherin protein [12]. Transgenic expression of *vtRNA1-1* in mice was not shown to alter baseline gut permeability in vivo. However, increases in gut permeability were seen during periods of vulnerability of the gut barrier, such as in response to the bacterial product lipopolysaccharide (LPS). Gut permeability increases appreciably more in vtRNA1-1Tg mice, compared with their littermates after exposure to identical doses of LPS. Treatment of littermate mice with a low dose of LPS (0.1 mg/kg) for 5 days fails to change gut permeability but induces markable gut barrier dysfunction in vtRNA1-1Tg mice.

Consistently, induction in the levels of mucosal *vtRNA2-1* by infecting mice with lenti-vtRNA2-1 also increases the vulnerability of the gut barrier under critical pathological conditions [10]. Exposure of mice to septic stress leads to an acute gut barrier dysfunction in both the lenti-vtRNA2-1-infected mice and mice infected with the control lentiviral vector. However, the increase in gut permeability in mice infected with lenti-vtRNA2-1 is much higher than that observed in mice infected with control vector after exposure to septic stress. In addition, increasing the levels of tissue *vtRNA2-1* or *vtRNA1-1* inhibits growth of the intestinal epithelium, as pointed out above, and this repression in intestinal mucosal growth may also contribute to *vtRNA*-mediated epithelial barrier dysfunction, since rapid and continuous intestinal epithelial renewal is crucial for maintaining the epithelial integrity that is essential for gut barrier structure and function. Together, the findings obtained from these in vitro, ex vivo, and in vivo experiments strongly support the concept that *vtRNAs* are the negative regulators of TJ expression and epithelial barrier function and that vtRNAs modulate the intestinal mucosal homeostasis by altering gut barrier function and constant epithelial renewal (Figure 2).

## 4. Interactions Between vtRNAs and RNA-Binding Proteins

The interactions of vtRNAs with proteins other than the vault proteins were established early when one group found a 50 kDa protein that often co-purified with the vault proteins and was found to interact with the vtRNA. This was later identified as the La RBP [61]. Several recent studies reveal that vtRNAs regulate intestinal epithelium homeostasis by interacting with RBPs, although the exact mechanism underlying vtRNA/RBP associations remains largely unknown. RBPs are a large family of proteins that directly interact with RNA transcripts throughout a cell’s RNA-driven processes and modulate stability and translation of target mRNAs positively or negatively [47,58,62]. The structures to which RBPs bind and mechanisms with which RBPs regulate RNAs are diverse. RBP interactions with various RNAs range from single protein-RNA element association to the formation of multiple RBPs and RNA molecules. RBPs have been increasingly recognized as a novel group of master posttranscriptional regulators in the intestinal epithelium homeostasis, whereas deregulation of RBPs and their interactions with ncRNAs disrupts integrity of the intestinal epithelium and plays an important role in the pathogenesis of different gut mucosal disorders such as IBD, delayed wound healing, infection, cancers, and sepsis [47,63,64].

### 4.1. vtRNA2-1 Interacts with HuR (ELAVL1)

HuR is a well-studied RBP that functions as an enhancer of mRNA stability and translation by binding with high affinity to the 3′-untranslated region (UTR) of target transcripts [65,66,67,68]. HuR is localized predominantly in the nucleus of unstimulated cells in the intestinal epithelium but can be rapidly translocated to the cytoplasm. It is in the cytoplasm where it can directly interact with target mRNAs in response to various cellular stressors, thereby altering gene expression levels. HuR interacts with the mRNAs encoding several TJ proteins including claudin-1, claudin-3, occludin and JAM-A, and AJ protein E-cadherin and increases the stability and translation of these target transcripts in the intestinal mucosa [58,69,70]. HuR associates with the 3′-UTR of the *occludin* mRNA and primarily promotes occludin translation but also has a minor effect on its mRNA stability. This interaction is tightly regulated by Chk2-dependent HuR phosphorylation [69,71] and another RBP, CUG-binding protein 1 (CUGBP1) [72]. HuR also up-regulates gap junction protein connexin 43 via stabilization of its mRNA in IECs [73] and activates autophagy by increasing ATG16L1 translation through interacting with circular RNA *circPABPN1* [64].

We have reported that *vtRNA2-1* represses translation of claudin-1 and occludin in the intestinal epithelium by decreasing HuR binding affinity for the TJ mRNAs [10]. *vtRNA2-1* directly binds to HuR in cultured IECs, since biotinylated *vtRNA2-1* specifically associates with HuR but does not bind to the RBP AU-binding factor 1 (AUF1), TJ proteins claudin-1, occludin, and E-cadherin, as determined by biotin pulldown analysis. Like *vtRNA1-1* [38], *vtRNA2-1* also binds to p62 in IECs. RNP immunoprecipitation (RIP) assays using anti-HuR and control IgG antibodies further reveal that *vtRNA2-1* is highly enriched in HuR immunoprecipitation (IP) materials compared to control IgG IP, indicating the association of endogenous *vtRNA2-1* with endogenous HuR in human Caco-2 cells. Increasing the level of cellular *vtRNA2-1* prevents HuR interaction with *claudin-1* and *occludin* mRNAs, since ectopically expressed *vtRNA2-1,* generated by transfecting cells with *vtRNA2-1* expression vector, almost totally blocks HuR binding to *claudin-1* mRNA and remarkably decreases the levels of HuR/*occludin* mRNA complex. On the other hand, increasing the levels of cellular *vtRNA2-1* does not affect total HuR abundance or its subcellular localization. Moreover, ectopically overexpressed HuR increases the levels of claudin-1 and occludin proteins, but this induction is abolished by increasing *vtRNA2-1* in cells overexpressing HuR. Increasing the levels of *vtRNA2-1* also prevents HuR binding to the *E-cadherin* mRNA and blocks HuR-induced expression of E-cadherin.

Importantly, the interaction between *vtRNA2-1* and HuR plays a critical role in epithelial barrier function regulation [10]. Induced HuR promotes the epithelial barrier function, as evidenced by increased TEER and decreased paracellular flux of FITC-dextran in HuR-overexpressed cells relative to control cells. However, this stimulation of epithelial barrier function by HuR is blocked by increasing *vtRNA2-1* since there are no significant differences in the levels of TEER and paracellular flux of FITC-dextran between cells co-transfected with HuR and *vtRNA2-1* expression vectors and cells transfected with control vector. Because HuR is a potent and biological stimulator of gut barrier function and its interaction with target mRNAs is highly regulated [47,69,72,74], these findings strongly suggest that increased *vtRNA2-1* impairs the epithelial barrier function at least partially by blocking HuR binding to the mRNAs encoding claudin-1, occludin, and E-cadherin, thereby inhibiting their expression.

### 4.2. vtRNA1-1 Interacts with CUGBP1

CUGBP1 binds to several different mRNA *cis*-elements, including GU-rich elements and AU-rich elements [57,64,75]. Interestingly, it was initially identified as an RBP, based on its binding ability to the short CUG repeats located in the DMPK gene’s 3′-UTR. The association of CUGBP1 with target mRNAs enhances mRNA decay and/or inhibits target transcript translation in general. CUGBP1 is highly expressed in the intestinal epithelium and negatively regulates TJ expression and gut barrier function [57,72]. Exposure of mice to septic stress increases levels of mucosal CUGBP1 in the small intestine, which is associated with an inhibition of TJ expression and gut barrier dysfunction [69,72]. CUGBP1 directly interacts with the mRNAs encoding occludin, claudin-1, and E-cadherin. However, it does not bind to the mRNAs encoding other membrane proteins such as claudin-2, claudin-3, claudin-5, ZO-1, and β-catenin. Interestingly, CUGBP1 and HuR compete for binding to the same occludin 3′-UTR resulting in competitive regulation of occludin translation [72]. CUGBP1 also inhibits renewal of the intestinal epithelium by repressing the translation of cyclin-dependent kinase 4 (CDK4) via interaction with both coding region and 3′-UTR of the *Cdk4* mRNA [4]. CUGBP1 activity is tightly controlled primarily by miR-222, miR-195, miR-503, HuR, and polyamines in the intestinal mucosa [4,76,77,78].

Our recent study reveals that *vtRNA1-1* regulates TJ expression and barrier function at least in part by its direct interaction with CUGBP1 [12]. Our results show that endogenous *vtRNA1-1* is associated with endogenous CUGBP1, as examined by RNP/RIP assays using an anti-CUGBP1 antibody. Both *vtRNA1-1* and *vtRNA2-1* are highly enriched in CUGBP1 IP compared to control. However, there are no noted differences in the total input vtRNA levels. This demonstrates an interaction between *vtRNA1-1* and CUGBP1 in IECs. Interaction between *vtRNA1-1* and CUGBP1 enhances CUGBP1 binding to the mRNAs encoding claudin-1 and occludin, since the levels of CUGBP1/*claudin-1* or CUGBP1/*occludin* mRNA complexes are greatly increased in cells overexpressing *vtRNA1-1*. Moreover, *vtRNA1-1* and CUGBP1 inhibit TJ protein expression synergistically, as evidenced by in vitro co-transfection studies. There was an additional decrease in the levels of certain TJ proteins like claudin-1 and occludin in cells co-transfected with both CUGBP1 and *vtRNA1-1* when compared with cells transfected with CUGBP1 alone. Importantly, CUGBP1-induced gut epithelial barrier dysfunction is increased by elevating the levels of cellular *vtRNA1-1*. Decreased TEER and increased paracellular flux of FITC-dextran are enhanced when the levels of both CUGBP1 and *vtRNA1-1* are increased by co-transfection, indicating the importance of interaction of *vtRNA1-1* with CUGBP1 in regulation of the gut barrier function.

### 4.3. vtRNAs Interact with p62 and Other RBPs

*vtRNA1-1* regulates selective autophagy through direct interaction with the RBP p62 by directly binding to autophagy receptor sequestosome-1/p62 in both mouse and human tissues [38]. Overexpression of *vtRNA1-1* leads to inhibition of p62-dependent autophagy, while decreasing the levels of *vtRNA1-1* by starvation leads to an increase in starvation-induced autophagy. The regulatory role of *vtRNA1-1* in autophagy is reliant on the ZZ and PB1 domains of p62 via the oligomerization patterns of p62 [79,80]. The PB1 domain as well as the adjacent region of p62 are necessary and sufficient for *vtRNA1-1* binding. There is a central flexible loop within *vtRNA1-1*, which is responsible for the *vtRNA1-1* interaction with p62 [81]. vtRNAs are also associated with the RBP hnRPN C that is localized in cytoplasm, and interaction between vtRNAs and hnRPN C are able to associate with viral RNA to promote viral replication [13].

**Figure 3:**
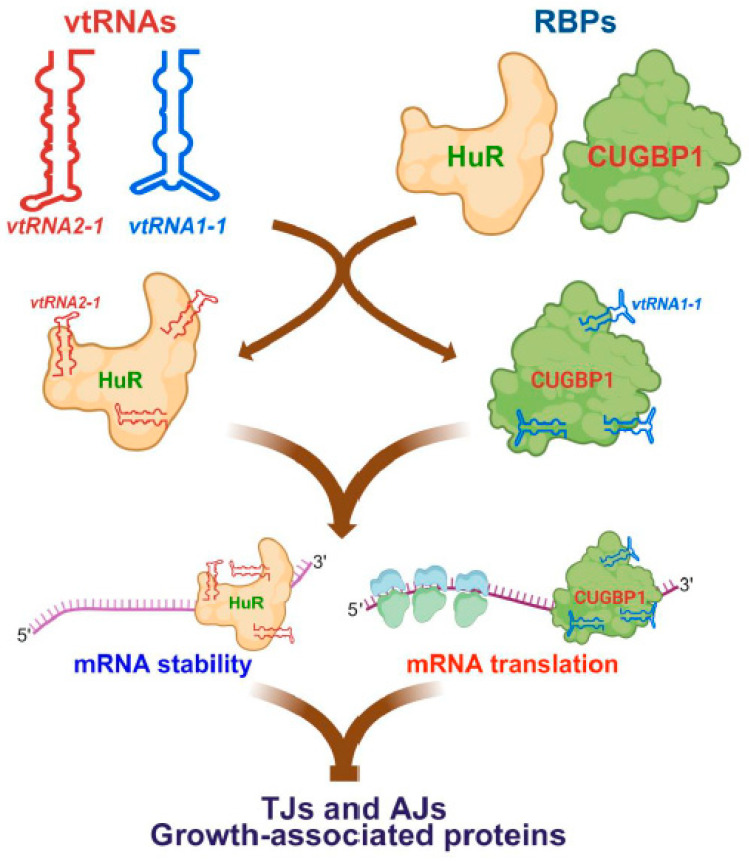
vtRNAs inhibit the expression of intercellular junctions at the posttranscriptional level through interactions with RNA-binding proteins (RBPs). *vtRNA1-1* interacts with CUGBP1 and enhances CUGBP1 binding affinity on target mRNAs, leading to a synergistic inhibition of mRNA stability and translation. In contrast, *vtRNA2-1* forms complex with HuR and this association inhibits HuR binding to given mRNAs, preventing HuR-mediated stimulation of target gene expression.

Taken together, we propose a model by which vtRNAs regulate gene regulatory programs governing gut barrier function and epithelial renewal by interacting with RBPs (Figure 3). CUGBP1 and HuR are highly expressed in the intestinal epithelium, and their cellular levels and binding affinity for certain target mRNAs are tightly modulated by many factors. Besides miRNAs, lncRNAs, circRNAs, and polyamines [7,47,57,78], interactions between vtRNAs and RBPs, particular formation of *vtRNA2-1*/HuR and *vtRNA1-1*/CUGPB1 complexes, affect binding affinity of RBPs for specific mRNAs and alter the stability and translation of target transcripts, thus contributing to the control of expression of TJs/AJs and proliferation-associated proteins in response to stressful environments. These exciting findings showing the association of vtRNAs with RBPs advance our understanding of vtRNAs and their biological function in intestinal epithelial homeostasis. It is also likely that the regulation of CUGBP1 and HuR by vtRNAs could represent a general mechanism underlying biological functions of RBPs in the gut epithelium.

## 5. Involvement of vtRNAs in Gut Mucosal Disorders and Other Diseases

As vtRNAs are intimately involved in many aspects of intestinal epithelial renewal and gut barrier function by interacting with RBPs, it is unsurprising that vtRNAs have been subsequently implicated in several gastrointestinal pathologies and human diseases.

### 5.1. Inflammatory Bowel Disease

One commonly seen and studied set of disorders of the intestinal tract is IBD, which is characterized by either mucosal, submucosal, or transluminal inflammatory injury/erosions of the bowel wall and can cause substantial morbidity among patients. IBD is made up of two similar but distinct diseases: ulcerative colitis (UC) and Crohn’s disease (CD). The colonic mucosal tissues from patients with active UC exhibit increased levels of *vtRNA2-1*, *vtRNA1-1*, and *vtRNA1-3* but not *vtRNA1-2*, compared to control tissues. On the other hand, only *vtRNA1-3* levels increase significantly in ileal mucosal tissues obtained from CD patients relative to control tissues, while the abundances of *vtRNA2-1, vtRNA1-1,* and *vtRNA1-2* in ileal mucosa from CD patients are identical to those observed in the mucosa from control individuals [10]. Consistent with the findings from IBD patients, colonic mucosal tissue from dextran sodium sulfate (DSS)-induced colitis in mice is also associated with a dramatical increase in *vtRNA* levels, achieving 6-fold higher levels in DSS-treated mice than those observed in control animals [10]. Interestingly, although mucosal growth was slowed in ex vivo organoid studies derived from transgenic mice with increased vtRNA1-1, the actual mice did not demonstrate changes to the colonic mucosal growth in vivo. This is potentially related to the slower mucosal turnover of the colon compared to the small bowel [12]. Although the exact role of altered vtRNAs in IBD remains unclear, these findings support the wide involvement of vtRNAs in human gut pathologies, since their expression levels in the intestinal epithelium change markedly in response to various critical stresses.

### 5.2. Critical Surgical Disorders

Acute injury to the epithelial lining of the intestine and gut barrier dysfunction are common in critical surgical disorders such as shock and sepsis, leading to translocation of luminal bacteria and toxic substances to the bloodstream. Several gut mucosa-enriched ncRNAs, including miRNAs and lncRNAs, have been implicated in disrupted homeostasis of the gut epithelium in patients with critical surgical disorders as summarized recently [45]. Interestingly, compared to extracellular vesicles (EVs) isolated from health controls, EVs isolated from the serum of shock patients are both more concentrated and contained more *vtRNA1-1* and *vtRNA2-1* [12]. In contrast, the levels of EV *vtRNA1-2* and *vtRNA1-3* are low in EVs and exhibit no significant differences between shock patients and control individuals. The levels of ‘free floating’ (non-EV-containing) vtRNAs in serum from shock patients and control subjects show no significant differences in the levels of any of the four human vtRNAs. A study examining the levels of other ncRNAs in EVs reveals that there are no meaningful differences in the levels of miR-29b, miR-195, and miR-222 in EVs between shock and control subject samples. The levels of lncRNAs *uc.173*, *uc.230*, *H19*, and *SPRY4-IT1* in EVs from both groups are below the limits of detection. Consistent with observations in humans, mice exposed to septic stress also exhibit increased serum levels of EV *vtRNA* and mucosal vtRNA in the small intestine, along with increased gut permeability [10,12]. These findings implicate vtRNAs in EVs and mucosal tissue in intestinal mucosal pathology in patients with critical surgical disorders.

### 5.3. Viral Infections

Several studies show that the levels of vtRNAs increase rapidly after exposure to viral infection and that an induction in vtRNAs favors viral replication [13,19,82]. Expression levels of vtRNAs increase during infection with various RNA as well as DNA viruses, specifically picornaviruses, alphaviruses, beta-coronaviruses, Epstein–Barr virus, and Kaposi’s sarcoma virus [13,19,83]. The expression of vtRNAs is also upregulated in lymphocytes following infection with various gamma-herpes viruses. Further investigation into this effect reveals that latent membrane protein 1 in EBV-infected B cells is the key factor causing upregulation of vtRNAs. Induced expression of *vtRNA1-1*, but not the other paralogues, improvs the establishment of the virus in the cells as well as reduces the apoptosis of infected cells via regulation of NF-κB cascade and RBPs such as HuR [13,84]. The expression of vtRNAs is also greatly increased in vitro and in a murine model, following infection with the influenza A virus. Silencing vtRNA leads to a decrease in viral replication in lung cells. Mechanistically, it appears that viruses can evade PKR-mediated innate immunity via vtRNAs, which are observed in several different viral infections [82].

### 5.4. Cancers

vtRNAs play an important role in pathogenesis of several cancers through control of cell proliferation, apoptosis, and autophagy [18,40,85,86,87,88]. For example, *vtRNA2-1* inhibits proliferation of several cancer cells, including colon, lung, breast, skin, cervical, and oral cancers, while *vtRNA1-1* regulates apoptosis and cancer invasion [37]. vtRNAs are also implicated in cellular resistance to mitoxantrone, a drug used to treat different types of cancers [89,90,91]. Following successful treatment of in vitro and in vivo endocrine tumors, there is an observed exosomal release of vtRNAs and subsequent upregulation of autophagy markers [92]. One mouse model demonstrated that the absence of vPARP and TEP1, which has been associated with reduced vtRNA stability, increased the animal’s susceptibility to chemical-indued colonic tumorigenesis [93]. Furthermore, there have been several studies looking at vtRNAs in different specific cancers [17,94,95,96,97]. These results from these studies clearly show that vtRNAs are intimately involved in many aspects of carcinogenesis of several cancers, including gastric cancer [95], esophageal squamous cell carcinoma [94,96], prostate cancer [60,98,99], hepatocellular carcinoma [39,100,101], hematopoietic malignancies and myelodysplastic syndrome [35,41,102,103,104], and endometrial cancer [36]. Components of the vault protein have additionally been implicated in certain cancers, including colorectal cancer, with MVP implicated in the migration of cancer cells and facilitation of metastasis [105]. The role of vtRNAs and their regulatory roles in various cancers has already been expertly reviewed, noting that not all of the 4 vtRNAs play the same roles in the same cancer processes [17].

## 6. Conclusions and Future Studies

Rapid renewal of the intestinal mucosa and effective gut barrier are essential for maintaining intestinal epithelium homeostasis and function. Impairment of mucosal growth and epithelial barrier function leads to the translocation of luminal pathogens into the bloodstream and promotes the progression of many critical diseases such as IBD, shock, sepsis, viral infection, and cancers. Regulation of mRNA stability and translation by ncRNAs and RBPs is a crucial process that affects gene expression at the posttranscriptional level and preserves the epithelial integrity in response to various pathophysiological stresses. Small noncoding vtRNAs are highly expressed in the intestinal epithelium and their tissue levels change dramatically in the mucosa with injury/erosions, inflammation, and infection in humans. The results summarized here provide clear evidence that vtRNAs, particularly altered *vtRNA1-1* and *vtRNA2-1*, play an important role in the regulation of intestinal mucosal renewal and epithelial barrier function. Although the mechanisms underlying vtRNAs in the intestinal epithelium homeostasis and diseases remain largely unknown, emerging results have demonstrated that vtRNAs negatively regulate intestinal mucosal adaptation and increase gut permeability primarily by interacting with RBPs HuR and CUGBP1.

However, there are still many gaps in our understanding of vtRNA functions in the intestinal epithelium and their potential clinical implications. First, a better understanding of vtRNA classification, modifications, and cellular effects in different situations is necessary and badly needed. Second, the pathobiological functions of various small vtRNAs via fragmentation in the intestinal epithelium homeostasis and their response to pathologic stress should be fully investigated, particularly in patients with critical illnesses. Third, we should define the mechanisms underlying control of the IEC proliferation and cell-to-cell interaction by vtRNAs, specifically their roles in mRNA stability and translation during normal as well as pathological states. While no specific diagnostic or therapeutic targets are clear at this time, we should search for molecular signatures that help clinical application using vtRNAs as biomarkers for acute gut barrier dysfunction in patients with critical surgical disorders and as potential therapeutic targets for developing novel therapeutics to protect the gut epithelium against injury and inflammation.

## Data Availability

No new data were created or analyzed in this study. Data sharing is not applicable to this article.

## Abbreviations

IECs	intestinal epithelial cells
ncRNAs	noncoding RNAs
lncRNAs	long ncRNAs
vtRNAs	vault RNAs
RBPs	RNA-binding proteins
IBD	inflammatory bowel diseases
UC	ulcerative colitis
CD	Crohn’s disease
miRNA	microRNA
UTRs	untranslated regions
circRNAs	circular RNAs
MVPs	major vault proteins
RNP	ribonucleoprotein
ISCs	intestinal stem cells
TEER	transepithelial electrical resistance
vtRNA1-1Tg	vtRNA1-1 transgenic mice
EVs	extracellular vesicles

## References

[1] Turner J.R. (2009). Intestinal mucosal barrier function in health and disease. Nat. Rev. Immunol..

[2] Yang H., Rao J.N., Wang J.-Y. (2014). Posttranscriptional regulation of intestinal epithelial tight junction barrier by RNA-binding proteins and microRNAs. Tissue Barriers.

[3] Bankaitis E.D., Ha A., Kuo C.J., Magness S.T. (2018). Reserve stem cells in intestinal homeostasis and injury. Gastroenterology.

[4] Xiao L., Cui Y.H., Rao J.N., Zou T., Liu L., Smith A., Turner D.J., Gorospe M., Wang J.Y. (2011). Regulation of cyclin-dependent kinase 4 translation through CUG-binding protein 1 and microRNA-222 by polyamines. Mol. Biol. Cell.

[5] Assimakopoulos S.F., Triantos C., Thomopoulos K., Fligou F., Maroulis I., Marangos M., Gogos C.A. (2018). Gut-origin sepsis in the critically ill patient: Pathophysiology and treatment. Infection.

[6] Ponting C.P., Oliver P.L., Reik W. (2009). Evolution and functions of long noncoding RNAs. Cell.

[7] Xiao L., Gorospe M., Wang J.Y. (2019). Long noncoding RNAs in intestinal epithelium homeostasis. Am. J. Physiol. Cell Physiol..

[8] Carninci P., Kasukawa T., Katayama S., Gough J., Frith M.C., Maeda N., Oyama R., Ravasi T., Lenhard B., Wells C. (2005). The transcriptional landscape of the mammalian genome. Science.

[9] Lander E.S., Linton L.M., Birren B., Nusbaum C., Zody M.C., Baldwin J., Devon K., Dewar K., Doyle M., FitzHugh W. (2001). Initial sequencing and analysis of the human genome. Nature.

[10] Ma X.X., Xiao L., Wen S.J., Yu T.X., Sharma S., Chung H.K., Warner B., Mallard C.G., Rao J.N., Gorospe M. (2023). Small noncoding vault RNA2-1 disrupts gut epithelial barrier function via interaction with HuR. EMBO Rep..

[11] Jouravleva K., Zamore P.D. (2025). A guide to the biogenesis and functions of endogenous small non-coding RNAs in animals. Nat. Rev. Mol. Cell Biol..

[12] Sharma S., Xiao L., Chung H.K., Chen T., Mallard C.G., Warner B., Yu T.X., Kwon M.S., Chae S., Raufman J.P. (2025). Noncoding Vault RNA1-1 Impairs Intestinal Epithelial Renewal and Barrier Function by Interacting With CUG-binding Protein 1. Cell. Mol. Gastroenterol. Hepatol..

[13] Stok J.E., van der Kooij S.B., Gravekamp D., Ter Haar L.R., de Wolf J.W., Salgado-Benvindo C., Nelemans T., Grijmans B.J.M., Tjokrodirijo R.T.N., de Ru A.H. (2025). Vault RNAs aid RNA virus infection by facilitating cytoplasmic localization of hnRNP C and ELAVL1. Cell Rep..

[14] Kedersha N.L., Rome L.H. (1986). Isolation and characterization of a novel ribonucleoprotein particle: Large structures contain a single species of small RNA. J. Cell Biol..

[15] Kedersha N.L., Miquel M.C., Bittner D., Rome L.H. (1990). Vaults. II. Ribonucleoprotein structures are highly conserved among higher and lower eukaryotes. J. Cell Biol..

[16] Aghajani Mir M. (2024). Vault RNAs (vtRNAs): Rediscovered non-coding RNAs with diverse physiological and pathological activities. Genes Dis..

[17] Taube M., Lisiak N., Toton E., Rubis B. (2024). Human vault RNAs: Exploring their potential role in cellular metabolism. Int. J. Mol. Sci..

[18] Kickhoefer V.A., Rajavel K.S., Scheffer G.L., Dalton W.S., Scheper R.J., Rome L.H. (1998). Vaults are up-regulated in multidrug-resistant cancer cell lines. J. Biol. Chem..

[19] Nandy C., Mrazek J., Stoiber H., Grasser F.A., Huttenhofer A., Polacek N. (2009). Epstein-barr virus-induced expression of a novel human vault RNA. J. Mol. Biol..

[20] Kong L.B., Siva A.C., Kickhoefer V.A., Rome L.H., Stewart P.L. (2000). RNA location and modeling of a WD40 repeat domain within the vault. RNA.

[21] van Zon A., Mossink M.H., Schoester M., Scheffer G.L., Scheper R.J., Sonneveld P., Wiemer E.A. (2001). Multiple human vault RNAs. Expression and association with the vault complex. J. Biol. Chem..

[22] Kickhoefer V.A., Liu Y., Kong L.B., Snow B.E., Stewart P.L., Harrington L., Rome L.H. (2001). The Telomerase/vault-associated protein TEP1 is required for vault RNA stability and its association with the vault particle. J. Cell Biol..

[23] Poderycki M.J., Rome L.H., Harrington L., Kickhoefer V.A. (2005). The p80 homology region of TEP1 is sufficient for its association with the telomerase and vault RNAs, and the vault particle. Nucleic Acids Res..

[24] Stadler P.F., Chen J.J.L., Hackermüller J., Hoffmann S., Horn F., Khaitovich P., Kretzschmar A.K., Mosig A., Prohaska S.J., Qi X. (2009). Evolution of vault RNAs. Mol. Biol. Evol..

[25] Kickhoefer V.A., Searles R.P., Kedersha N.L., Garber M.E., Johnson D.L., Rome L.H. (1993). Vault ribonucleoprotein particles from rat and bullfrog contain a related small RNA that is transcribed by RNA polymerase III. J. Biol. Chem..

[26] Kickhoefer V.A., Emre N., Stephen A.G., Poderycki M.J., Rome L.H. (2003). Identification of conserved vault RNA expression elements and a non-expressed mouse vault RNA gene. Gene.

[27] Kolev N.G., Rajan K.S., Tycowski K.T., Toh J.Y., Shi H., Lei Y., Michaeli S., Tschudi C. (2019). The vault RNA of Trypanosoma brucei plays a role in the production of trans-spliced mRNA. J. Biol. Chem..

[28] Zavrelova A., Shen S., Zahedifard F., Agbebi E.A., Braune S., Kramer S., Zoltner M. (2025). The trypanosome vault particle is composed of multiple major vault protein paralogs and harbors vault RNA. J. Biol. Chem..

[29] Hussain S., Sajini A.A., Blanco S., Dietmann S., Lombard P., Sugimoto Y., Paramor M., Gleeson J.G., Odom D.T., Ule J. (2013). NSun2-mediated cytosine-5 methylation of vault noncoding RNA determines its processing into regulatory small RNAs. Cell Rep..

[30] Cao J., Song Y., Bi N., Shen J., Liu W., Fan J., Sun G., Tong T., He J., Shi Y. (2013). DNA methylation-mediated repression of miR-886-3p predicts poor outcome of human small cell lung cancer. Cancer Res..

[31] Dugue P.A., Yu C., McKay T., Wong E.M., Joo J.E., Tsimiklis H., Hammet F., Mahmoodi M., Theys D., kConFab (2021). VTRNA2-1: Genetic variation, Heritable Methylation and Disease Association. Int. J. Mol. Sci..

[32] Hahne J.C., Lampis A., Valeri N. (2021). Vault RNAs: Hidden gems in RNA and protein regulation. Cell. Mol. Life Sci..

[33] Avila-Bonilla R.G., Martinez-Montero J.P. (2024). Crosstalk between vault RNAs and innate immunity. Mol. Biol. Rep..

[34] Kunkeaw N., Jeon S.H., Lee K., Johnson B.H., Tanasanvimon S., Javle M., Pairojkul C., Chamgramol Y., Wongfieng W., Gong B. (2013). Cell death/proliferation roles for nc886, a non-coding RNA, in the protein kinase R pathway in cholangiocarcinoma. Oncogene.

[35] Helbo A.S., Treppendahl M., Aslan D., Dimopoulos K., Nandrup-Bus C., Holm M.S., Andersen M.K., Liang G., Kristensen L.S., Gronbaek K. (2015). Hypermethylation of the VTRNA1-3 promoter is associated with poor outcome in lower risk myelodysplastic syndrome patients. Genes.

[36] Hu Z., Zhang H., Tang L., Lou M., Geng Y. (2017). Silencing nc886, a non-coding RNA, induces apoptosis of human endometrial cancer cells-1A in vitro. Med. Sci. Monit..

[37] Bracher L., Ferro I., Pulido-Quetglas C., Ruepp M.D., Johnson R., Polacek N. (2020). Human vtRNA1-1 levels modulate signaling pathways and regulate apoptosis in human cancer cells. Biomolecules.

[38] Horos R., Buscher M., Kleinendorst R., Alleaume A.M., Tarafder A.K., Schwarzl T., Dziuba D., Tischer C., Zielonka E.M., Adak A. (2019). The small non-coding vault RNA1-1 acts as a riboregulator of autophagy. Cell.

[39] Ferro I., Gavini J., Gallo S., Bracher L., Landolfo M., Candinas D., Stroka D.M., Polacek N. (2022). The human vault RNA enhances tumorigenesis and chemoresistance through the lysosome in hepatocellular carcinoma. Autophagy.

[40] Lee K., Kunkeaw N., Jeon S.H., Lee I., Johnson B.H., Kang G.Y., Bang J.Y., Park H.S., Leelayuwat C., Lee Y.S. (2011). Precursor miR-886, a novel noncoding RNA repressed in cancer, associates with PKR and modulates its activity. RNA.

[41] Prajapat M., Sala L., Vidigal J.A. (2024). The small noncoding RNA Vaultrc5 is dispensable to mouse development. RNA.

[42] Yu T.X., Kalakonda S., Liu X., Han N., Chung H.K., Xiao L., Rao J.N., He T.C., Raufman J.P., Wang J.Y. (2022). Long noncoding RNA uc.230/CUG-binding protein 1 axis sustains intestinal epithelial homeostasis and response to tissue injury. JCI Insight.

[43] Kwon M.S., Chung H.K., Xiao L., Yu T.X., Sharma S., Cairns C.M., Chen T., Chae S., Turner D.J., Wang J.Y. (2024). Interaction between microRNA-195 and HuR regulates Paneth cell function in the intestinal epithelium by altering SOX9 translation. Am. J. Physiol. Cell Physiol..

[44] Wang J.Y., Xiao L., Wang J.Y. (2017). Posttranscriptional regulation of intestinal epithelial integrity by noncoding RNAs. Wiley Interdiscip. Rev. RNA.

[45] Cairns C.A., Xiao L., Wang J.Y. (2024). Posttranscriptional regulation of intestinal mucosal growth and adaptation by noncoding RNAs in critical surgical disorders. J. Investig. Surg..

[46] Xiao L., Li X.X., Chung H.K., Kalakonda S., Cai J.Z., Cao S., Chen N., Liu Y., Rao J.N., Wang H.Y. (2019). RNA-binding protein hur regulates paneth cell function by altering membrane localization of TLR2 via post-transcriptional control of CNPY3. Gastroenterology.

[47] Sharma S., Xiao L., Wang J.Y. (2023). HuR and its interactions with noncoding RNAs in gut epithelium homeostasis and diseases. Front. Biosci. Landmark.

[48] Beumer J., Clevers H. (2021). Cell fate specification and differentiation in the adult mammalian intestine. Nat. Rev. Mol. Cell Biol..

[49] Xiao L., Warner B., Mallard C.G., Chung H.K., Shetty A., Brantner C.A., Rao J.N., Yochum G.S., Koltun W.A., To K.B. (2023). Control of Paneth cell function by HuR regulates gut mucosal growth by altering stem cell activity. Life Sci. Alliance.

[50] Lindemans C.A., Calafiore M., Mertelsmann A.M., O’Connor M.H., Dudakov J.A., Jenq R.R., Velardi E., Young L.F., Smith O.M., Lawrence G. (2015). Interleukin-22 promotes intestinal-stem-cell-mediated epithelial regeneration. Nature.

[51] Chung H.K., Xiao L., Han N., Chen J., Yao V., Cairns C.M., Raufman B., Rao J.N., Turner D.J., Kozar R. (2024). Circular RNA Cdr1as inhibits proliferation and delays injury-induced regeneration of the intestinal epithelium. JCI Insight.

[52] Chung H.K., Xiao L., Jaladanki K.C., Wang J.Y. (2021). Regulation of paneth cell function by RNA-binding proteins and noncoding RNAs. Cells.

[53] Lueschow S.R., McElroy S.J. (2020). The Paneth Cell: The Curator and Defender of the Immature Small Intestine. Front. Immunol..

[54] Blanco S., Kurowski A., Nichols J., Watt F.M., Benitah S.A., Frye M. (2011). The RNA-methyltransferase Misu (NSun2) poises epidermal stem cells to differentiate. PLoS Genet..

[55] Sajini A.A., Choudhury N.R., Wagner R.E., Bornelov S., Selmi T., Spanos C., Dietmann S., Rappsilber J., Michlewski G., Frye M. (2019). Loss of 5-methylcytosine alters the biogenesis of vault-derived small RNAs to coordinate epidermal differentiation. Nat. Commun..

[56] Alagia A., Terenova J., Ketley R.F., Di Fazio A., Chelysheva I., Gullerova M. (2023). Small vault RNA1-2 modulates expression of cell membrane proteins through nascent RNA silencing. Life Sci. Alliance.

[57] Chung H.K., Rao J.N., Wang J.-Y. (2022). Regulation of Gut Barrier Function by RNA-Binding Proteins and Noncoding RNAs.

[58] Xiao L., Rao J.N., Wang J.Y. (2021). RNA-binding proteins and long noncoding RNAs in intestinal epithelial autophagy and barrier function. Tissue Barriers.

[59] Chelakkot C., Ghim J., Ryu S.H. (2018). Mechanisms regulating intestinal barrier integrity and its pathological implications. Exp. Mol. Med..

[60] Fort R.S., Garat B., Sotelo-Silveira J.R., Duhagon M.A. (2020). vtRNA2-1/nc886 produces a small RNA that contributes to its tumor suppression action through the microRNA pathway in prostate cancer. Non-Coding RNA.

[61] Kickhoefer V.A., Poderycki M.J., Chan E.K., Rome L.H. (2002). The La RNA-binding protein interacts with the vault RNA and is a vault-associated protein. J. Biol. Chem..

[62] Gebauer F., Schwarzl T., Valcarcel J., Hentze M.W. (2021). RNA-binding proteins in human genetic disease. Nat. Rev. Genet..

[63] Chatterji P., Rustgi A.K. (2018). RNA binding proteins in intestinal epithelial biology and colorectal cancer. Trends Mol. Med..

[64] Li X.X., Xiao L., Chung H.K., Ma X.X., Liu X., Song J.L., Jin C.Z., Rao J.N., Gorospe M., Wang J.Y. (2020). Interaction between HuR and circPABPN1 modulates autophagy in the intestinal epithelium by altering ATG16L1 translation. Mol. Cell. Biol..

[65] Abdelmohsen K., Pullmann R., Lal A., Kim H.H., Galban S., Yang X., Blethrow J.D., Walker M., Shubert J., Gillespie D.A. (2007). Phosphorylation of HuR by Chk2 regulates SIRT1 expression. Mol. Cell.

[66] Brennan C.M., Steitz J.A. (2001). HuR and mRNA stability. Cell. Mol. Life Sci..

[67] Giammanco A., Blanc V., Montenegro G., Klos C., Xie Y., Kennedy S., Luo J., Chang S.H., Hla T., Nalbantoglu I. (2014). Intestinal epithelial HuR modulates distinct pathways of proliferation and apoptosis and attenuates small intestinal and colonic tumor development. Cancer Res..

[68] Lebedeva S., Jens M., Theil K., Schwanhausser B., Selbach M., Landthaler M., Rajewsky N. (2011). Transcriptome-wide analysis of regulatory interactions of the RNA-binding protein HuR. Mol. Cell.

[69] Yu T.X., Wang P.Y., Rao J.N., Zou T., Liu L., Xiao L., Gorospe M., Wang J.Y. (2011). Chk2-dependent HuR phosphorylation regulates occludin mRNA translation and epithelial barrier function. Nucleic Acids Res..

[70] Xiao L., Rao J.N., Cao S., Liu L., Chung H.K., Zhang Y., Zhang J., Liu Y., Gorospe M., Wang J.Y. (2016). Long noncoding RNA SPRY4-IT1 regulates intestinal epithelial barrier function by modulating the expression levels of tight junction proteins. Mol. Biol. Cell.

[71] Xu Y., Tian Y., Wang Y., Yang J., Li F., Wan X., Ouyang M. (2021). Human antigen R (HuR) and cold inducible RNA-binding protein (CIRP) influence intestinal mucosal barrier function in ulcerative colitis by competitive regulation on Claudin1. Biofactors.

[72] Yu T.X., Rao J.N., Zou T., Liu L., Xiao L., Ouyang M., Cao S., Gorospe M., Wang J.Y. (2013). Competitive binding of CUGBP1 and HuR to occludin mRNA controls its translation and modulates epithelial barrier function. Mol. Biol. Cell.

[73] Wang S.R., Mallard C.G., Cairns C.A., Chung H.K., Yoo D., Jaladanki S.K., Xiao L., Wang J.Y. (2023). Stabilization of Cx43 mRNA via RNA-binding protein HuR regulated by polyamines enhances intestinal epithelial barrier function. Am. J. Physiol. Gastrointest. Liver Physiol..

[74] Chung H.K., Wang S.R., Xiao L., Rathor N., Turner D.J., Yang P., Gorospe M., Rao J.N., Wang J.Y. (2018). alpha4 coordinates small intestinal epithelium homeostasis by regulating stability of HuR. Mol. Cell. Biol..

[75] Qin W.J., Shi J.J., Chen R.Y., Li C.Y., Liu Y.J., Lu J.F., Yang G.J., Cao J.F., Chen J. (2024). Curriculum vitae of CUG binding protein 1 (CELF1) in homeostasis and diseases: A systematic review. Cell. Mol. Biol. Lett..

[76] Cui Y.H., Xiao L., Rao J.N., Zou T., Liu L., Chen Y., Turner D.J., Gorospe M., Wang J.Y. (2012). miR-503 represses CUG-binding protein 1 translation by recruiting CUGBP1 mRNA to processing bodies. Mol. Biol. Cell.

[77] Zhang Y., Zhang Y., Xiao L., Yu T.X., Li J.Z., Rao J.N., Turner D.J., Gorospe M., Wang J.Y. (2017). Cooperative repression of insulin-like growth factor type 2 receptor translation by microRNA 195 and RNA-binding protein CUGBP1. Mol. Cell. Biol..

[78] Rao J.N., Xiao L., Wang J.Y. (2020). Polyamines in Gut Epithelial Renewal and Barrier Function. Physiology.

[79] Buscher M., Horos R., Hentze M.W. (2020). ‘High vault-age’: Non-coding RNA control of autophagy. Open Biol..

[80] Johansen T. (2019). Selective Autophagy: RNA comes from the vault to regulate p62/SQSTM1. Curr. Biol..

[81] Buscher M., Horos R., Huppertz I., Haubrich K., Dobrev N., Baudin F., Hennig J., Hentze M.W. (2022). Vault RNA1-1 riboregulates the autophagic function of p62 by binding to lysine 7 and arginine 21, both of which are critical for p62 oligomerization. RNA.

[82] Li F., Chen Y., Zhang Z., Ouyang J., Wang Y., Yan R., Huang S., Gao G.F., Guo G., Chen J.L. (2015). Robust expression of vault RNAs induced by influenza A virus plays a critical role in suppression of PKR-mediated innate immunity. Nucleic Acids Res..

[83] Mrazek J., Kreutmayer S.B., Grasser F.A., Polacek N., Huttenhofer A. (2007). Subtractive hybridization identifies novel differentially expressed ncRNA species in EBV-infected human B cells. Nucleic Acids Res..

[84] Amort M., Nachbauer B., Tuzlak S., Kieser A., Schepers A., Villunger A., Polacek N. (2015). Expression of the vault RNA protects cells from undergoing apoptosis. Nat. Commun..

[85] Lei J., Xiao J.H., Zhang S.H., Liu Z.Q., Huang K., Luo Z.P., Xiao X.L., Hong Z.D. (2017). Non-coding RNA 886 promotes renal cell carcinoma growth and metastasis through the Janus kinase 2/signal transducer and activator of transcription 3 signaling pathway. Mol. Med. Rep..

[86] Gao W., Zhang S., Guorong L., Liu Q., Zhu A., Gui F., Zou Y., Wu Y., Luo Y., Hong Z. (2022). Nc886 promotes renal cancer cell drug-resistance by enhancing EMT through Rock2 phosphorylation-mediated beta-catenin nuclear translocation. Cell Cycle.

[87] Gallo S., Kong E., Ferro I., Polacek N. (2022). Small but Powerful: The human vault RNAs as multifaceted modulators of pro-survival characteristics and tumorigenesis. Cancers.

[88] Horos R., Büscher M., Sachse C., Hentze M.W. (2019). Vault RNA emerges as a regulator of selective autophagy. Autophagy.

[89] Gopinath S.C., Matsugami A., Katahira M., Kumar P.K. (2005). Human vault-associated non-coding RNAs bind to mitoxantrone, a chemotherapeutic compound. Nucleic Acids Res..

[90] Berger W., Steiner E., Grusch M., Elbling L., Micksche M. (2009). Vaults and the major vault protein: Novel roles in signal pathway regulation and immunity. Cell. Mol. Life Sci..

[91] Gopinath S.C., Wadhwa R., Kumar P.K. (2010). Expression of noncoding vault RNA in human malignant cells and its importance in mitoxantrone resistance. Mol. Cancer Res..

[92] Bornstein S., Shapiro I., Mazumdar A., Zitzmann K., Nolting S., Luca E., Beuschlein F., Sharma A., Hantel C. (2023). The vault complex is significantly involved in therapeutic responsiveness of endocrine tumors and linked to autophagy under chemotherapeutic conditions. Cancers.

[93] Raval-Fernandes S., Kickhoefer V.A., Kitchen C., Rome L.H. (2005). Increased susceptibility of vault poly(ADP-ribose) polymerase-deficient mice to carcinogen-induced tumorigenesis. Cancer Res..

[94] Lee H.S., Lee K., Jang H.J., Lee G.K., Park J.L., Kim S.Y., Kim S.B., Johnson B.H., Zo J.I., Lee J.S. (2014). Epigenetic silencing of the non-coding RNA nc886 provokes oncogenes during human esophageal tumorigenesis. Oncotarget.

[95] Lee K.S., Park J.L., Lee K., Richardson L.E., Johnson B.H., Lee H.S., Lee J.S., Kim S.B., Kwon O.H., Song K.S. (2014). nc886, a non-coding RNA of anti-proliferative role, is suppressed by CpG DNA methylation in human gastric cancer. Oncotarget.

[96] Dai W., Liu H. (2022). MicroRNA-886 suppresses osteosarcoma cell proliferation and its maturation is suppressed by long non-coding RNA OXCT1-AS1. Bioengineered.

[97] Kong L., Hao Q., Wang Y., Zhou P., Zou B., Zhang Y.X. (2015). Regulation of p53 expression and apoptosis by vault RNA2-1-5p in cervical cancer cells. Oncotarget.

[98] Fort R.S., Matho C., Geraldo M.V., Ottati M.C., Yamashita A.S., Saito K.C., Leite K.R.M., Mendez M., Maedo N., Mendez L. (2018). Nc886 is epigenetically repressed in prostate cancer and acts as a tumor suppressor through the inhibition of cell growth. BMC Cancer.

[99] Oliveira-Rizzo C., Colantuono C.L., Fernández-Alvarez A.J., Boccaccio G.L., Garat B., Sotelo-Silveira J.R., Khan S., Ignatchenko V., Lee Y.S., Kislinger T. (2025). Multi-Omics Study Reveals Nc886/vtRNA2-1 as a Positive Regulator of Prostate Cancer Cell Immunity. J. Proteome Res..

[100] Yu M.C., Lee C.W., Lin C.H., Wu C.H., Lee Y.S., Tsai C.L., Tsai C.N. (2020). Differential hypermethylation of the VTRNA2-1 promoter in hepatocellular carcinoma as a prognostic factor: Tumor marker prognostic study. Int. J. Surg..

[101] Kong E., Polacek N. (2025). TRIM21 modulates stability of pro-survival non-coding RNA vtRNA1-1 in human hepatocellular carcinoma cells. PLoS Genet..

[102] Treppendahl M.B., Qiu X., Sogaard A., Yang X., Nandrup-Bus C., Hother C., Andersen M.K., Kjeldsen L., Mollgard L., Hellstrom-Lindberg E. (2012). Allelic methylation levels of the noncoding VTRNA2-1 located on chromosome 5q31.1 predict outcome in AML. Blood.

[103] Kato H., Hashimoto Y., Hatayama Y., Shimohiro H., Motokura T. (2023). Serum levels of vault RNA significantly varied in patients with haematological malignancies. Mol. Med. Rep..

[104] Hatayama Y., Shimohiro H., Hashimoto Y., Ichikawa H., Kawamura K., Motokura T. (2025). Serum vault RNA1-1 levels reflect blood cells and bone marrow. Mol. Cell. Probes.

[105] Pietras P., Leśniczak-Staszak M., Kasprzak A., Andrzejewska M., Jopek K., Sowiński M., Rucinski M., Lyons S.M., Ivanov P., Szaflarski W. (2021). MVP Expression Facilitates Tumor Cell Proliferation and Migration Supporting Metastasis of Colorectal Cancer Cells. Int. J. Mol. Sci..

